# Outcome of match related allogeneic stem cell transplantation procedures performed from 2004 till 2011

**DOI:** 10.1186/2162-3619-1-13

**Published:** 2012-05-18

**Authors:** Natasha Ali, Salman Naseem Adil, Mohammad Usman Shaikh, Munira Moosajee, Nehal Masood

**Affiliations:** 1 FCPS Haematology, Department of Pathology and Microbiology, The Aga Khan University and Hospital, Karachi, Pakistan; 2 Department of Medicine, The Aga Khan University and Hospital, Karachi, Pakistan; 3 Department of Pathology and Microbiology, The Aga Khan University Hospital, P.O Box 3500, Stadium Road, Karachi, 74800, Pakistan

**Keywords:** Outcome, Allogeneic transplant, Overall survival, Allogeneic transplant, GvHD, Overall survival

## Abstract

We present our initial experience of allogeneic stem cell transplant procedure performed between April 2004 and August 2011 for various haematological disorders. All patients with non-malignant and malignant haematological disorders with HLA matched donors were selected after pre-transplant workup. Ninety seven patients underwent the procedure. Most common indications for transplant were aplastic anaemia in n = 34 (35%), followed by β-Thalassemia major in n = 21 (21.6%) and chronic myeloid leukemia in n = 11 patients (11.3%). Primary graft failure present was present in 2.06%. Incidence of graft versus host disease (GvHD) in our patients was 34%. After median follow-up of five years the overall survival was 71.3% with a mean survival time of 51.2 ± 3.3 months.

## Introduction

Haematopoietic stem cell transplant is now the standard of care in many congenital or acquired, malignant and non-malignant haematological diseases. The last few years have seen rapid increases in volumes of the procedure. In 2006, approximately 60,000 transplants were performed worldwide [1]. Although the history of transplant began in the late 1940s and 1950s when animal studies revealed the ability of donor bone marrow to restore hematopoieis after irradiation, in Pakistan, stem cell transplant was started in 1995[2].

In a country like ours where consanguinity prevails, β- thalassemia major is the most common genetic haematological disorder requiring stem cell transplant as a curative treatment option [3]. Pakistan is also included in the list of countries where prevalence of aplastic anaemia is high. This is second most common indication for transplant in our setting [4]. Apart from these two disorders, rest of the allogeneic procedures mainly revolve around chronic and acute leukaemia. Due to the large family sizes, in 70% of the patients an HLA identical sibling donor is available as compared to the western population. Autologous stem cell transplant is mainly indicated in lymphomas and myeloma but frequency is lower as compared to allogeneic procedures.

With a per capita income of $1051 (2009–2010), the affordability of stem cell transplant procedure by an average man is prohibited by its cost which ranges from $100,000 -$150,000. Although the cost is cheaper locally, majority of our transplants are funded mainly by nongovernmental organisations and philanthropists.

Currently, stem cell transplant is being performed in three centres. Our centre was established in April 2004. Initially it was a two bedded unit which was upgraded to four bedded in 2006. With this background, we present our initial experience between April 2004 and August 2011 of allogeneic haematopoietic stem cell transplant for various haematological disorders.

## Subjects and methods

All patients with non-malignant and malignant haematological disorders with HLA matched donors were selected for the procedure.

### **Pre-transplant work-up**

Complete blood counts, liver and kidney function tests, and infectious disease profile (consisting of hepatitis B surface antigen, hepatitis C antibody, HIV antibody, Cytomegalovirus, Mantoux test, chest and dental roentgenograms), along with blood grouping and coagulation testing was performed in all donors. For patients, screening included all the above mentioned investigations along with pulmonary function tests, echocardiography and dental evaluation.

### **Stem cell mobilization**

All donors were given granulocyte–colony stimulating factor (G-CSF) at a dose of 5-10 μg/kg/twice daily for five days prior to harvest. Patients with donors less than five years received bone marrow only as the stem cell source. In patients with aplastic anaemia, peripheral blood as well as bone marrow stem cells were the preferred source. In all other conditions peripheral blood progenitor cells only were used as the source of stem cells.

### **Conditioning regimen**

Patients with Thalassemia, Acute Myeloid Leukemia, Chronic Myeloid Leukemia, Biphenotypic Leukemia and Philadelphia negative Acute Lymphoblastic Leukemia received Busulfan (4 mg/day for four days) and Cyclophosphamide (60 mg/kg/day for two days) as conditioning chemotherapy. Class III thalassemic patients received conditioning with hyperchelation protocol which consisted of deferoxamine 40 mg/kg, hydroxyurea 30 mg/kg and azathioprine 3 mg/kg daily between day-45 and day-11 before transplantation. From day-17 till day-13, Fludarabine was administered at a dose of 20 mg/m^2^/day. On day-10, Busulfan was started at 1 mg/kg thrice daily for four days (total 14 doses) followed by cyclophosphamide 40 mg/kg for four days [5]. Total body irradiation (1.5cGY x twice a day) and Cyclophosphamide (60 mg/kg/day for two days) was used in patients with Philadelphia positive Acute Lymphoblastic Leukemia and those with one-antigen mismatch donors.

In Aplastic anaemia **rabbit** anti-thymocyte globulin (10 mg/kg/day for three days) and Cyclophosphamide (50 mg/kg/day for four days) was used. Patients with Fanconi’s anaemia received conditioning with **Fludarabine (30 mg/kg/day for five days), Cyclophosphamide (300 mg/m**^**2**^**for four days) and rabbit anti-thymocyte globulin (3.75 mg/kg/day for three days)**.

### **Infectious disease prophylaxis**

Patients were admitted in protective isolation equipped with HEPA filter, positive pressure and laminar airflow ventilation. Standard prophylaxis with Ciprofloxacin (500 mg twice daily or 20-30 mg/kg/two divided doses), Fluconazole (200 mg once daily or 6 mg/kg/day) and Valaciclovir (500 mg twice daily or 10 mg/kg/twice daily) was started in all patients on day-5. All patients were provided with neutropenic diet.

### **Graft versus host disease prophylaxis**

Intravenous Cyclosporine was started on day-1 and doses were adjusted according to drug levels. Optimum adult range was 200-250 ng/dl. For paediatric patients, levels were maintained between 150-200 ng.dl. Methotrexate 15 mg/m^2^ was administered on day +1, while 10 mg/m^2^ was given on days +3 +6. Irradiated and leukocyte reduced blood products were used throughout admission as well as in the post-transplant period

### **Assessment of engraftment**

Absolute neutrophil count of ≥500 x 10^9^/L for three consecutive days was considered engraftment in all patients. For further confirmation, post-transplant chimerism studies of patient and donor were carried out at day + 90.

### **Statistical analysis**

All the data was entered on SPSS version 19 (SPSS Inc., Chicago, IL, USA) for computing means, standard deviation and range of all descriptive variables. Survival was calculated from the date of transplant to death or last follow-up according to Kaplan-Meier analysis methods.

## Results

From April 2004 till September 2011, n = 97 patients underwent allogeneic stem cell transplant procedure for various haematological disorders. Four patients received stem cells from complete matched parent donor while all others had a HLA compatible sibling donor. There were n = 70 (72.2%) males and n = 27 (27.8%) females. N = 44 (45.4%) patients belonged to the paediatric population while n = 53 (54.6%) were adults. Median age ± SD was 17 years ±12.5 years (range: 2–53 years).

**Figure 1:**
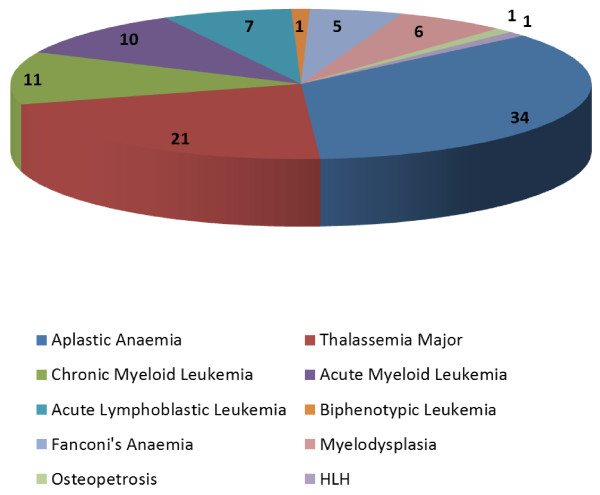
Indications for allogeneic stem cell transplant.

Most common indications for transplant were idiopathic aplastic anaemia in n = 34 (35%), followed by β-Thalassemia major in n = 21 (21.6%) and chronic myeloid leukemia in n = 11 patients (Figure 1). For acute lymphoblastic leukemia, n = 4 were positive for Philadelphia chromosome, n = 3 had disease relapse. Three patients with acute myeloid leukemia (AML) were in complete remission at the time of transplant. Procedure for relapsed AML was done in n = 5 patients. There were n = 2 patients with high risk disease (one patient developed AML preceded by myelodysplastic syndrome while one had Monosomy 8). At the time of admission, n = 4 patients (4.1%) were hepatitis C reactive. All four patients were suffering from severe aplastic anaemia therefore hepatitis C treatment at that point in time was not possible due to thrombocytopenia.

Seventeen patients (17.5%) received only bone marrow as a source of stem cells. Peripheral blood was used in n = 50 patients (51.5%) while n = 30 (30.9%) received both bone marrow and peripheral blood. Primary graft failure was seen in 2.06%. All other patients (n = 95; 98%) achieved successful engraftment. Median time ± SD to neutrophil recovery was 13 ± 3.1 days (range: 9–22 days) while for platelet recovery was 17 ± 3.4 days (range: 13 to 36 days). Secondary graft failure occurred in n = 2 patients with aplastic anaemia and n = 1 patient with β-thalassemia major. Seven patients with Aplastic anaemia expired due to E-Coli sepsis (n = 6) and Fusarium infection (n = 1).

**Figure 2:**
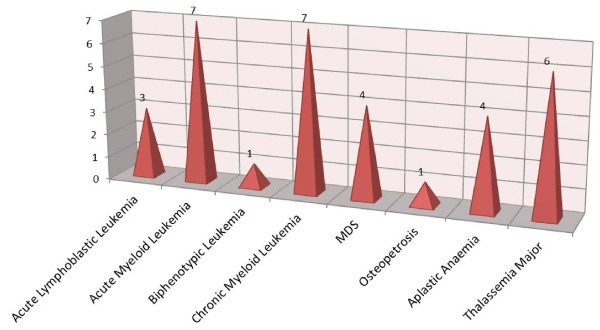
Incidence of GvHD according to diagnosis.

Overall incidence of graft versus host disease (GvHD) in our patients was 34%. Out of these, n = 18 (18.6%) developed acute GvHD (grade I-IV) whereas n = 15 (15.5%) had chronic GvHD (limited –n = 2 and extensive – n = 13). Frequency of GvHD according to diagnosis is shown in Figure 2.

Other post-transplant non infective complications were veno-occlusive disease in n = 4 (4.1%), haemorrhagic cystitis in n = 2 (2.06%), cyclosporine induced thrombotic microangiopathy in n = 1 patient and venous thromboembolism in n = 2 patients. In one of the patient with haemorrhagic cystitis, the cause was BK virus infection. He responded to supportive treatment resulting in complete recovery.

Other post-transplant infective complications included fungal infections in n = 2 patients, herpes zoster in n = 6 patients and cytomegalovirus infection in n = 1 patient.

Transplant related mortality was seen in n = 14 patients. Causes of mortality included sepsis in n = 8 patients. Mortality due to single organism infection (E coli) was seen in n = 4 and Fusarium was seen in n = 1 patient. Others were infected with multiple organisms which included E Coli and Pseudomonas (n = 1) Ecoli, acinetobacter and alcaligenes (n = 2). Other causes were fulminant hepatic failure (1), adult respiratory distress syndrome (1), graft failure (1), GvHD (2) and conditioning related mortality (1).

**Figure 3:**
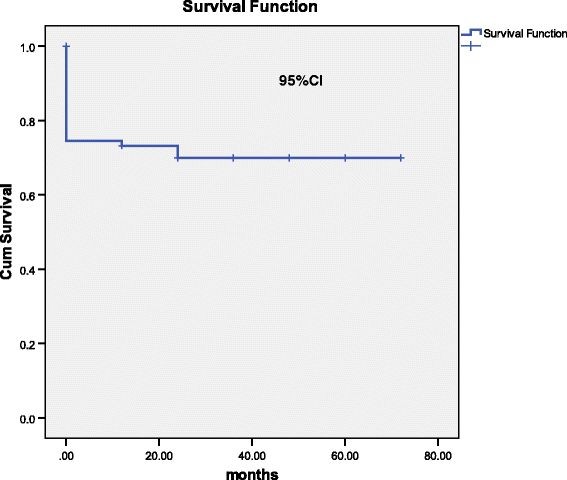
Overall survival in allogeneic stem cell transplant n=97.

**Figure 4:**
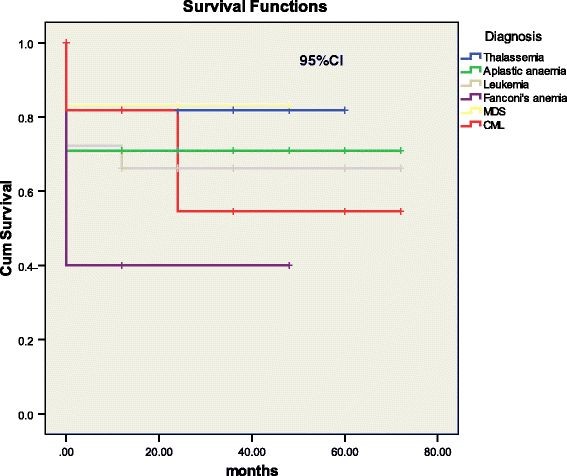
Overall survival according to diagnosis n=97.

At the end of 5 years, the overall survival (OS) of allogeneic transplant in our study was 71.3% with a mean survival time of 51.2 ± 3.3 months. In Aplastic anaemia the overall survival was 71% with a mean survival time of 51 ± 5.8 months. For β thalassemia major patients, the mean survival time was 49 ± 4.9 months and overall survival was 81.8%. In chronic myeloid leukemia, OS was 63.6% with a mean survival time of 45.8 ± 9.9 months. Lastly, in patients with acute leukemia, the overall survival was 66.7%. The disease wise survivals are shown in Figure 3 and 4.

## Discussion

For many congenital and acquired haematological disorders, haematopoietic stem cell transplant has become an established procedure. The European group for Blood and Bone Marrow transplantation survey in 2009 reported 41% allogeneic transplants from six hundred and twenty four centres (43 countries). The main indications were leukemias, lymphomas, solid tumours and non-malignant disorders [6]. These results are in sharp contrast with our centre where we have done allogeneic transplants in non-malignant disorders like aplastic anaemia and β-Thalassemia major.

The Eastern Mediterranean Bone Marrow Transplantation group has reported acute leukemias, bone marrow failure syndromes and haemoglobinopathies as the most common indications [7]. A similar study done in 2007 from our country also shows results compatible with ours [8]. The most prevalent genetically transmitted haematological disorder in Pakistan is β- Thalassemia major with a carrier rate of 5-8%. Around 5,000 children are diagnosed each year [9] and safe transfusion practices along with iron chelation therapy are very expensive in our country therefore transplant remains to be the only available curative option.

Currently, peripheral blood cells are increasingly replacing bone marrow as a source of stem cells in allogeneic stem cell transplant[10].We have also followed the same protocol except in aplastic anaemia where combination of bone marrow as well as peripheral blood progenitor cells was used in order to decrease the incidence of graft versus host disease.

Primary graft failure after haematopoietic stem cell transplantation is a life threatening complication, the reported incidence being as high as 11% [11]. Our study has reported an incidence of 2% which is much lower when compared to international literature. The largest study to date done by Schriber J et al. [12] quotes highest frequency in match unrelated donor transplants. No donor registry exists in our country therefore at the moment we are performing match related transplants as a result of which we have decreased incidence of graft failure.

Graft versus host disease remains to be one of the major complications of allogeneic bone marrow transplant. Despite HLA identity between patient and donor approximately 40% of recipients develop acute systemic GvHD [13]. None of our patients who received bone marrow only as a source of stem cells developed GvHD. We are not using T-cell depleted grafts therefore the incidence was highest with using only peripheral blood as a source of stem cells. Nevertheless, data from our centre has shown a lower incidence of GvHD as compared to national and international literature.

Neutropenia in post-transplant patients gives rise to bacterial infections. The percentage of gram negative organisms causing blood stream infections has decreased to 30% in the last two decades. Gram positive organisms now attribute to 70% of infections [14]. However, in our part of the world the trend remains unchanged as major pathogens are still gram negative bacteria most commonly E coli seen in our study as well as other studies from our country.

The survival of patients with beta thalassemia major has improved with regular blood transfusions and iron chelation therapy. However, definitive long term cure has been established through stem cell transplant. The centre with the largest experience is that of Lucarelli and his colleagues at Pesaro, Italy where survival has been reported as 73% [15]. Our study including all three groups of thalassemic patients has also shown similar results.

The recommended treatment for idiopathic aplastic anaemia is allogeneic stem transplant provided an HLA matched sibling donor is available. Survival rates have been reported as high as 85-90%. Wang W et al. has reported 74.1% [16] while Eapen M et al. [17] have reported an overall survival of 76% by using bone marrow only as a source of stem cells. In our group of patients, bone marrow as well as peripheral blood was used as a combination for the source of stem cells with 71% survival which is equivalent to international literature.

Chen Y et al. in 2011 reported an overall survival of 73.4% in n = 104 patients undergoing stem cell transplants for haematological malignancies [18]. An overall survival of 48% was found in patients undergoing allogeneic stem cell transplant in first complete remission (CR1) for acute myeloid leukemia. Our study shows a similar figure (OS: 50%) in AML. However, our cohort consisted of patients undergoing transplants in CR1 as well relapsed acute myeloid leukemia.

Historically, allogeneic stem cell transplant for acute lymphoblastic leukemia (ALL) in CR1 has been reserved for patients with high risk disease, such as those with Philadelphia positive ALL (ph + ve ALL) [19]. Our cohort included n = 4 patients with ph + ve ALL while n = 3 patients had T-cell ALL. The overall survival in these patients was 71.4%. However the overall survival in n = 4 ph + ve patients was 75%. Although the sample size is small, this frequency is similar to GOELAMS trial [20] but inferior to other studies conducted for ALL.

Haemopoietic stem cell transplant in chronic myeloid leukemia is now mostly indicated for patients who develop resistance to tyrosine kinase inhibitors [21]. Boehm A et al. in 2011 has reported an overall survival of 62% with a better outcome seen in chronic phase CML as compared to accelerated CML [22]. In our group of n = 11 patients, 2 patients had accelerated CML while others were in the chronic phase. The overall survival however, is in concordance with the compared study i.e. 63.6%.

Overall survival in our cohort was 71%. More than half of the transplants were performed for non-malignant diseases like Thalassemia Major and Aplastic Anaemia (n = 55). The median age of these n = 55 patients was 18 ± 12.6 years (range: 2–53 years). Therefore, a better overall survival in our patients can be attributed to transplant for non-malignant diseases, match related stem cells and younger age group.

The government of Pakistan has spent 0.6-1.1% of its GDP and 5.1% - 11.6% of its developmental expenditure on health over the last 10 years. The account of private sector health services is not included in this figure. Pakistan’s GDP in 2010 was reported as $161.99 billion (23) and average household income ranges from $1200-$1500 per annum. The cost of standard uncomplicated stem cell transplant ranges from $21,000 - $26,000, a figure affordable by handful of patients therefore n = 68 (70%) of our transplants were funded completely and/or partially by the hospital, non-governmental organisations and philanthropists.

## Conclusion

Allogeneic stem cell transplant remains to be the only curative therapy in many potentially fatal haematological diseases and is now being offered as a treatment option in a developing country like ours. Aplastic anaemia and β-thalassemia major were the common indications for the procedure. Frequency of acute and chronic GvHD was lower as compared to international literature. The overall survival in our study was approximately 71%.

## Consent

Written informed consent was obtained for allogeneic transplant from all patients.

## Competing interests

The authors declare that they have no competing interests.

## Authors’ contributions

NA collected data, drafted manuscript, performed statistical analysis. SNA, US, MM and NM provided critical review and helped in drafting manuscript. All authors read and approved the final manuscript.
